# Non-syndromic Oligodontia in Primary Dentition: A Report of a Rare Case

**DOI:** 10.7759/cureus.39426

**Published:** 2023-05-24

**Authors:** Sheetal Badnaware, Vinay Kumar Srivastava, Meenakshi Chandel

**Affiliations:** 1 Department of Pedodontics and Preventive Dentistry, Faculty of Dental Sciences, Institute of Medical Sciences, Banaras Hindu University, Varanasi, IND

**Keywords:** msx-1 and pax-1 gene, missing teeth, oligodontia, hypodontia, primary teeth

## Abstract

The congenital absence of teeth is the most common dental anomaly affecting 2.2% to 10% of the population. It could be present in the form of anodontia, hypodontia, or oligodontia, excluding wisdom teeth. Oligodontia is most commonly associated with several syndromes like ectodermal dysplasia, Down syndrome, and Van der Woude syndrome that involve the mutation of the MSX-1 and PAX-1 genes. Few cases have been reported in the literature on how oligodontia affects primary dentition. In this case report, a total of 17 primary teeth were missing. This case report investigates whether the features of non-syndromic oligodontia are present in the primary dentition in a two-year-old boy.

## Introduction

The congenital absence of teeth is the most prevalent developmental anomaly [1]. This condition has the following three forms: anodontia, hypodontia, and oligodontia. Anodontia is characterized by the complete absence of all teeth, while hypodontia is defined as the absence of six or fewer teeth and oligodontia entails the absence of six or more teeth [1,2]. The latter two are extremely rarely seen in primary dentition. The prevalence rate of hypodontia in primary dentition is 0.1%-0.9% [3]. The incisor region is most affected by hypodontia or oligodontia with congenitally missing succedaneous teeth, which may be due to ectodermal mucosal defects [4]. Oligodontia is inherited as an autosomal dominant pattern with incomplete penetrance of the traits and a variable expression. The cause of agenesis of the teeth is believed to be either genetic or hereditary or due to trauma, radiation overdose, glandular dysfunction, a systemic condition, or a syndrome [4]. However, non-syndromic oligodontia in primary dentition is rarely reported in the literature. This case report presents the case of a non-syndromic child with the clinical and radiological features of a two-year-old boy with missing primary teeth.

## Case presentation

**Figure 1:**
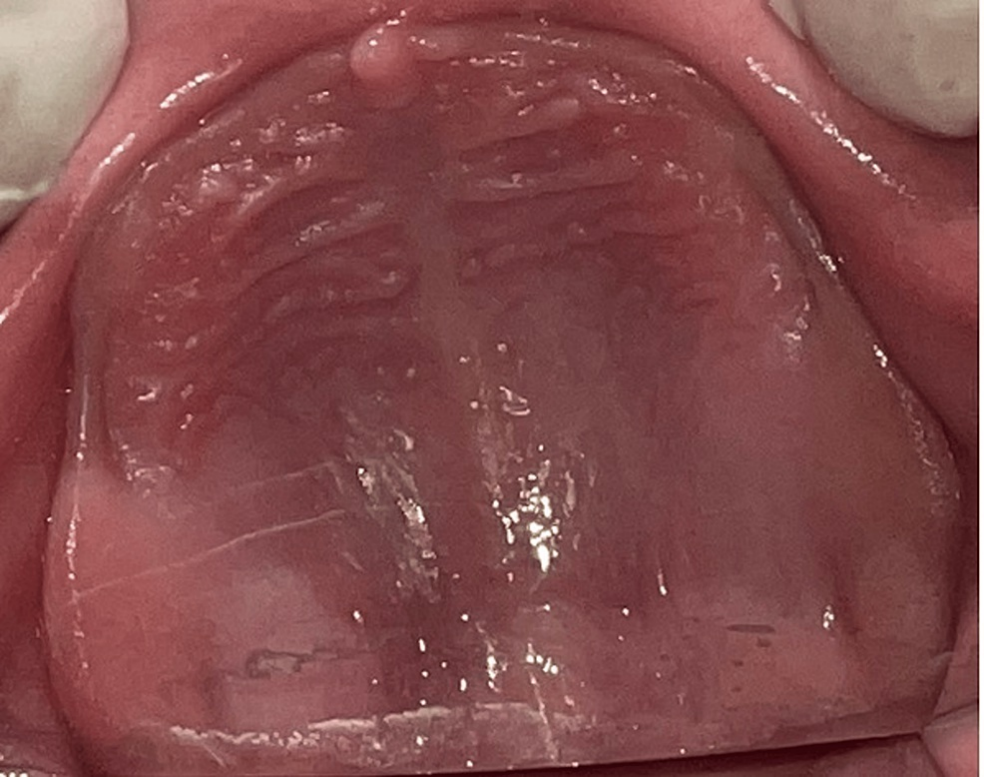
Maxillary intraoral view showing the absence of all primary teeth

**Figure 2:**
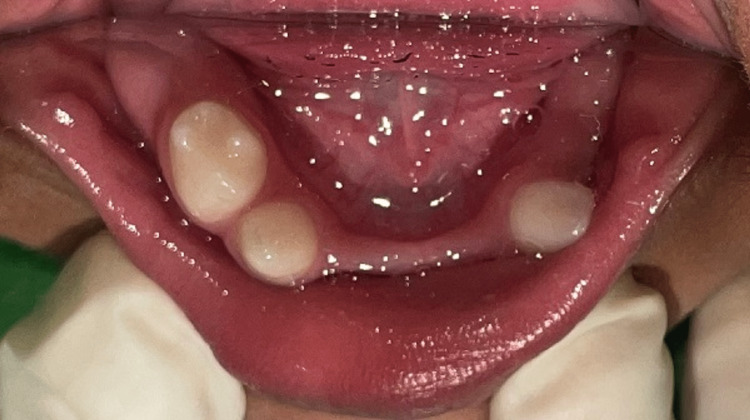
Mandibular intraoral view showing the absence of all primary teeth except 83, 84, and 73

**Figure 3:**
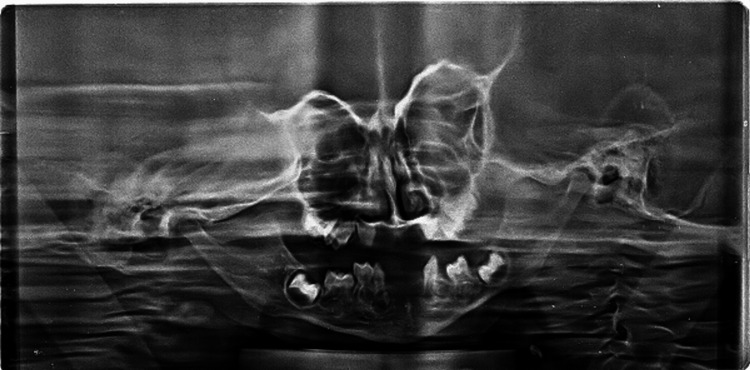
Orthopantomogram (OPG) view showing the presence of tooth buds 83, 84, 85, 46, 73, 75, 36, 16, 53, 55, 63, 65, and 26 and the absence of other tooth buds

**Figure 4:**
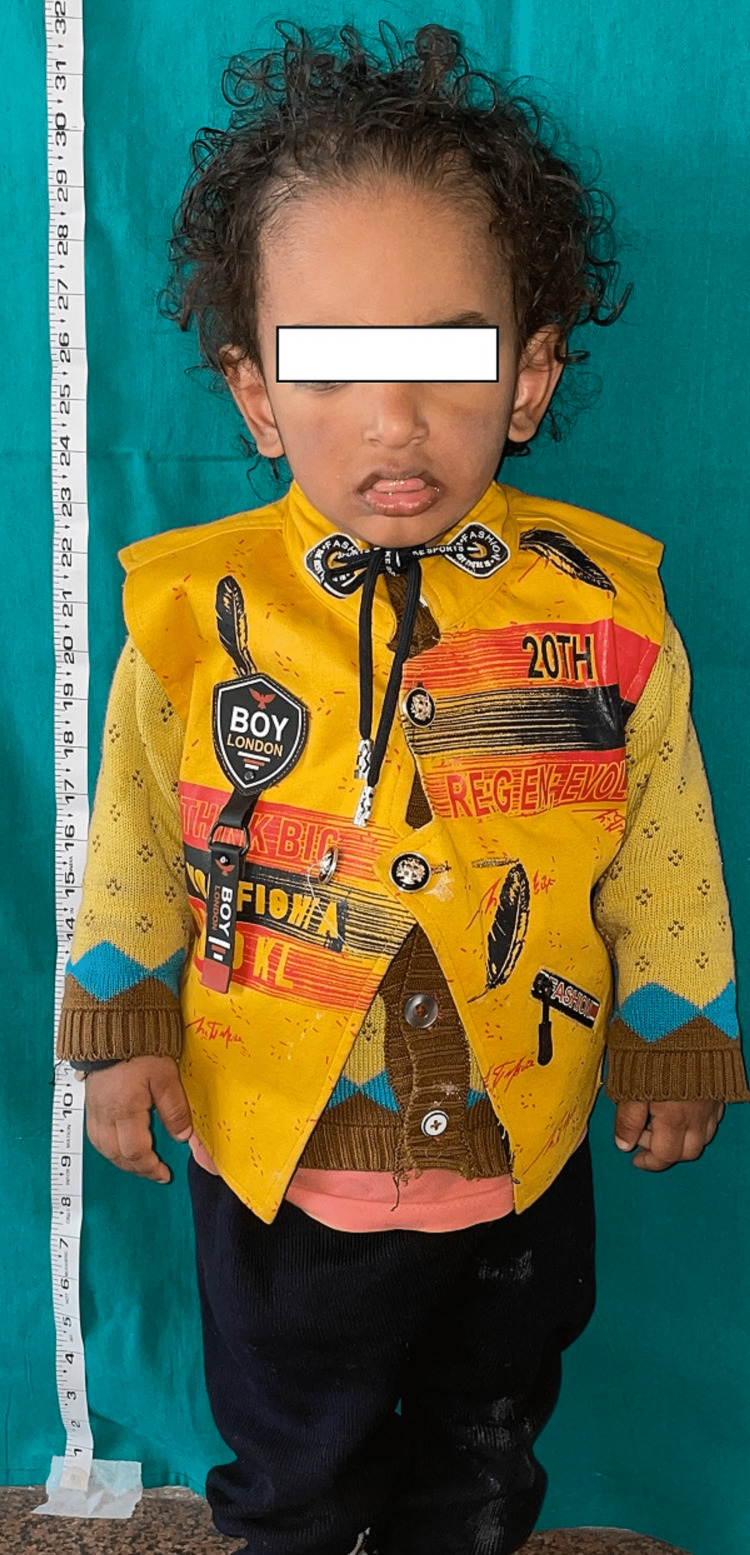
Extraoral view showing a depressed nasal bridge, straight profile, brachycephalic head, and normal height

A two-year-old boy reported to the unit of pedodontics and preventive dentistry with a chief complaint of missing teeth. The child was of Indian origin and was born from a non-consanguineous marriage with premature cesarean delivery in the seventh month. He was the only child of his parents. His father was a businessman, and his mother was a homemaker. They were both non-alcoholics, non-smokers and did not have any significant medical history. His family history was not contributory. During the patient’s first visit, an intraoral examination revealed that 17 primary teeth were absent in the oral cavity. In the maxillary arch, all primary teeth were absent clinically (Figure 1), and in the mandibular arch, only 83, 84, and 73 were present (Figure 2). The pre-cooperative nature of the child made it challenging to take an orthopantomogram (OPG). In the radiological examination, it was difficult to identify specific teeth due to the abnormal eruption pattern and shape of the tooth buds. The OPG revealed the presence of tooth buds 83, 84, 85, 46, 73, 75, 36, 16, 53, 55, 63, 65, and 26 and the absence of other tooth buds (Figure 3). All developmental milestones had been achieved [5]. The child was breastfed for one year and never bottle-fed. The height of the child was 30 inches (76 cm), and his weight was 10.7 kg, indicating a normal growth parameter. The child was a mouth breather. The extraoral examination indicated a depressed nasal bridge, straight profile, and a brachycephalic head, and the ectodermal features involving the skin, hair, and nails were normal (Figure 4). A complete set of investigations was conducted by a pediatrician, including measuring the serum levels of calcium, phosphate, T3, T4, and TSH (thyroid-stimulating hormone), and the complete blood count was within the normal limits, except the serum alkaline phosphatase level, which was high. The chromosomal karyotyping examination was also normal for a male (46XY). A diagnosis of non-syndromic oligodontia was made.

## Discussion

The congenital absence of permanent teeth is a common finding, but agenesis of primary teeth is rare [4]. Nevertheless, finding the cause of missing teeth, particularly in primary dentition, can be challenging for the clinician. In the literature, it is evident that the etiology of oligodontia could be influenced by genetic, epigenetic, or environmental factors and host factors such as chemotherapy, radiotherapy, and metabolic imbalances [6]. The most common causes of agenesis noted in the literature are associated with mutations in the gene, such as MSX1, EDA, and PAX9 [6,7]. The most commonly missing permanent tooth is the permanent maxillary lateral incisor, followed by the mandibular second premolar, excluding wisdom teeth [8]. In the primary dentition, the most commonly affected teeth in hypodontia are the primary maxillary lateral incisor and mandibular central incisors with a prevalence of <1% [9]. Oligodontia has a prevalence of between 0.5% and 0.9% and may occur alone or as a part of a syndrome or in more serious systemic disturbances [10]. The case report presented in this paper is highly interesting for several reasons. First, in the literature, very few cases of non-syndromic oligodontia in primary dentition associated with a normal growth parameter have been reported. The first primary teeth are visible on the radiographs during the 16th week of intrauterine (IU) life, and at the 26th IU life, two cusps of the primary first molar, one cusp of the primary second molar, and a crypt of the permanent first molar are visible. Schour and Massler (1961) introduced a chart explaining the eruption pattern of teeth [11]. They studied the development of the sequence of dentition in several stages using both radiographical and histological methods. According to this chart, at the age of 2( ± 6 months) years, clinically, all primary teeth should have erupted in the oral cavity, and calcification of permanent teeth, i.e., central and lateral incisors, canines, first premolars, and molars of all sides (a total of 20 tooth buds), should be seen radiographically [11]. Nirmala et al. Shashikiran et al., and Daugaard-Jensen et al. published case reports in which the total number of missing primary teeth was 14, 9, and 14, respectively [4,8,12]. In our case report, clinically 17 primary teeth were missing. They could either erupt later or be missing congenitally. The characteristics findings in this case report, which are the height and weight of the child, are within the normal ranges. According to the growth chart of the Indian Academy of Paediatrics, the height at the age of two years should be 80-92 cm, and the weight should be 9-14 kg [13]. All possible causes of this case have been ruled out by the pediatrician, including chromosomal karyotyping. The chromosomal karyotyping was normal for a child at 46XY. This is a classic case of non-syndromic oligodontia in primary dentition, which is rarely seen. Non-syndromic oligodontia can be sporadic or familial, and the inheritance pattern could be autosomal dominant, autosomal recessive, or X-linked [14]. The differential diagnosis of the non-syndromic form of oligodontia is established based on a thorough physical examination involving the nails, hair, sweat gland, eyes, and any skeletal and congenital disorders [4]. The associative syndromes of oligodontia include ectodermal dysplasia, Down syndrome, Rieger syndrome, Van der Woude syndrome, cleft lip and palate, oral-facial-digital syndrome, hemifacial microsomia, and Marshall syndrome. Al Shahrani et al. conducted a systematic review on hypodontia that suggested that genetics or heredity is the main etiology [3]. Jorgenson suggested that the physical obstruction of dental lamina was an etiology factor of oligodontia in oral-facial-digital syndrome [15]. Environmental factors, such as virus infection, radiotherapy or chemotherapy, and toxins, may cause the absence of permanent teeth, which is not present in our case report. Therefore, careful evaluation of all findings should be considered when planning the treatment. The oral rehabilitation of such young patients with missing teeth depends upon the growth and development of the individual patient. Careful monitoring and a long-term follow-up are required to avoid speech and aesthetic issues, as well as the development of deleterious oral habits.

## Conclusions

This case report described the importance of radiography as a diagnostic tool for different age estimations and the role of pediatric dentists in the early diagnosis and clinical evaluation of growth and development. A correct diagnosis in the early stage can rule out the possibility of syndromic and non-syndromic oligodontia or hypodontia. Oligodontia should not be neglected at any stage, and pediatric dentists should aim to treat it as soon as possible.
